# Animal Models Used in Hepatitis C Virus Research

**DOI:** 10.3390/ijms21113869

**Published:** 2020-05-29

**Authors:** Keith A. Berggren, Saori Suzuki, Alexander Ploss

**Affiliations:** Department of Molecular Biology, Princeton University, Princeton, NJ 08540, USA; keithab@princeton.edu (K.A.B.); stamura@princeton.edu (S.S.)

**Keywords:** hepatitis C, hepatitis C virus, animal model, host tropism, humanized mice

## Abstract

The narrow range of species permissive to infection by hepatitis C virus (HCV) presents a unique challenge to the development of useful animal models for studying HCV, as well as host immune responses and development of chronic infection and disease. Following earlier studies in chimpanzees, several unique approaches have been pursued to develop useful animal models for research while avoiding the important ethical concerns and costs inherent in research with chimpanzees. Genetically related hepatotropic viruses that infect animals are being used as surrogates for HCV in research studies; chimeras of these surrogate viruses harboring specific regions of the HCV genome are being developed to improve their utility for vaccine testing. Concurrently, genetically humanized mice are being developed and continually advanced using human factors known to be involved in virus entry and replication. Further, xenotransplantation of human hepatocytes into mice allows for the direct study of HCV infection in human liver tissue in a small animal model. The current advances in each of these approaches are discussed in the present review.

## 1. The Continuing Impact of HCV and the Utility of Animal Models

Globally, over 71 million people [1] are chronically infected with hepatitis C virus (HCV), resulting in greatly increased risk of developing liver fibrosis, decompensated cirrhosis and hepatocellular carcinoma (HCC). HCV is prevalent in all geographic regions, with numbers still increasing annually, especially in at-risk groups who are less likely to have access to diagnostic care or treatment, such as injection-drug users [1,2]. The lack of an effective vaccine contributes to the spread of infection, with an estimated 1.75 million new cases of chronic HCV infection per year [1].

The state of HCV treatment has changed rapidly in recent years, as direct-acting antiviral (DAA) drug therapy has gained widespread use, with over 90% of treated individuals clearing the virus entirely with the newest treatment regimens [3,4,5,6]. From the 1990s through the 2000s, HCV treatments advanced, with rates of HCV clearance in in patients rising from ~5% to ~40–80% as combination ribavirin/interferon treatments were accepted for widespread use [4]. Direct-acting antivirals began to be accepted for use in HCV patients in the mid-2000s and as they have largely supplanted use of combined interferon-based therapies, rates of HCV virus clearance have risen steadily in treated patients [4]. These drugs target the viral NS3/4 serine protease, the NS5A phosphoprotein and the NS5B RNA-dependent polymerase, inhibiting their respective functions in viral polyprotein processing and replication [5,6,7]. While these drugs have greatly advanced our ability to treat HCV infections, the high cost and limited availability of these drugs across the world and in disadvantaged or at-risk populations continues to be a major barrier [8,9,10]. In particular, access to these treatments is unavailable to resource-poor environments with less developed health care systems, patients without sufficient health coverage in countries without funded national health care programs, and injection drug users and abusers at higher risk for reinfection [9,11,12,13]. The continued burden of this disease in these populations, as well as the high cost of treatment, shows the need for a vaccine for HCV.

The continuing opioid crisis has contributed to an increase in HCV infection within the United States in recent years [14]. While this increase is troubling, HCV has long been a prevalent cause of chronic hepatitis leading to severe liver damage, and therefore efforts to develop a vaccine have been ongoing. Even in individuals who have access to DAA treatments, or gain access later, the well-documented liver damage resulting from chronic HCV infection persists, and increases risk of morbidities from development of liver fibrosis and hepatocellular carcinoma [10,15]. A prophylactic, such as a vaccine, shows great promise to mitigate the liver damage which occurs from undiagnosed or untreated chronic HCV infection, even with a highly effective treatment available. Efforts to develop a vaccine have been held back by the lack of an animal model that recapitulates both human HCV infection and the human immune response. An incomplete understanding of relevant protective immune responses further hinders these efforts [16,17]. Recently, only one vaccine candidate has progressed into clinical safety, immunogenicity and efficacy trials (NCT01436357) [18]. This candidate’s early stage clinical trial progression was based on immunogenicity studies in small non-human primates (NHPs) [19] and efficacy studies in chimpanzees [20], which can no longer be conducted due to the moratorium on the use of chimpanzees for biomedical research [21].

Globally, increased restrictions are in place on the use of chimpanzees in biomedical research; the official inclusion of captive chimpanzees on the U.S. Fish and Wildlife Service’s Endangered Species list has also precluded all use of these animals in future HCV research in the United States [21]. The need for a new animal model for early phase testing, as well as the rising incidence and prevalence of infection, highlights the need for continued research on HCV. This need is compounded by the inaccessibility of the most effective treatments to those most at risk of infection, and there is an urgent call for development of prophylactics and for a deeper understanding of the replication cycle of HCV. Because HCV robustly infects only humans and chimpanzees, more complex approaches have been taken in the development of small animal models of infection. Some work has focused on modification of mice to allow HCV infection, while other advances have been made in identifying and modifying viruses with similarity to HCV to be used as surrogates for study of hepatotropic infection and disease. This approach allows for production of infection and immune responses in small animal models, with utility for advancing our understanding of the mechanisms relevant to HCV. In the subsequent sections, we will discuss the progress that has been made to develop different (small) animal models with susceptibility to HCV or these closely related surrogate viruses.

## 2. HCV Infection in Non-Human Primates

Since HCV was first isolated and classified as non-A, non-B hepatitis virus from a patient in 1989 by Choo et al. [22,23], only two natural hosts have been found: humans and chimpanzees. This is a critical obstacle that hinders the study of HCV. Although chimpanzees are naturally susceptible to HCV infection, there are currently no reports of viral RNA found in wild chimpanzees. It is presently unknown whether other great ape species can support HCV infection or serve as natural reservoirs of the virus.

### 2.1. Chimpanzee Use for Study of HCV Infection

Historically, the use of chimpanzees in HCV research has greatly advanced our understanding of its infectious course. In 1997, an important breakthrough was the development of infectious HCV molecular clones, which were infectious in chimpanzees despite no production of viral particles in cell culture [24,25]. From this discovery until 2011 when chimpanzee research was banned in most countries, chimpanzees were the most important in vivo model for HCV study [26]. Chimpanzees had previously been a major component of research on this virus, possessing the potential to evaluate vaccine candidates due to their close genetic relatedness to humans; we possess greater than 98% genetic similarity. Furthermore, use of chimpanzees allowed straightforward study of immune responses and viral kinetics in the early acute phase of HCV infection. In human infections, patients rarely recognize symptoms of infection before development of acute hepatitis, and infections are often missed altogether when patients are asymptomatic [27]. Chimpanzee studies have contributed to our fundamental understanding of the molecular, immunological and clinical aspects of HCV infection.

Although susceptible to infection, the chronicity rate and symptoms of infection in chimpanzees are slightly different than in humans infected with HCV. Disease in HCV-infected chimpanzees appears to be generally mild; acute experimental infections resolve spontaneously at a much higher rate than in humans. Unlike in humans where approximately 75%–85% of patients progress to chronic hepatitis, the development of chronic disease after HCV infection in chimpanzees has been recorded between 33% and >60% across studies. While this shows variation in chronic hepatitis development in chimpanzee infections, it remains uniformly and significantly lower than the rate of chronic disease development in human infections [28,29,30]. Related to this low level of chronic infection development, only one case of hepatocellular carcinoma (HCC) has been reported in chimpanzees [31]. The severity of acute HCV infection is generally attenuated in chimpanzees as well. Currently, it remains incompletely understood why chimpanzees more readily clear acute infection with significantly milder disease rates and outcomes. Elucidating what causes these differences may prove to be integral to developing HCV vaccines. As previously reviewed [27,32], markers of innate immune response activation, including interferon alpha and related responses, are upregulated in the liver at the early acute phase of infection [33,34,35,36]. Notably, the levels of these virus-induced responses do not always correlate with clearance of HCV [35,36].

Humoral immune responses to HCV also appear to differ between humans and chimpanzees. It has been reported that levels of antibodies against HCV envelope proteins do not rise as rapidly in chimpanzees and do not reach the same levels that are found in human patients [37,38]. Further, animals with acute resolution of infection are not seen to develop neutralizing antibodies, whereas animals with persistent infection mount humoral immune responses [39]. This implies that lower levels of neutralizing antibodies in chimpanzees correlate with lower levels of viral replication during the early acute phase, leading to acute resolution. Conversely, in patients who spontaneously clear an acute HCV infection, levels of broadly neutralizing antibodies are even higher than those in persistently infected patients, where neutralizing antibody production is delayed before progressive broadening of antibody reactivity [40,41].

Chimpanzee studies have also contributed greatly to our understanding of cellular immune responses during the acute phase of HCV infection. Chimpanzees with acute clearance of infection had vigorous intrahepatic HCV-specific CD8^+^ T cell responses, whereas those with prolonged infection had much weaker responses [27,42]. Seminal studies demonstrated that depletion of CD8^+^ T cells resulted in prolonged HCV infection and CD4^+^ T cell response during early phase infection is associated with clearance [43,44].

### 2.2. Use of New World Monkeys and Old World Monkeys

These advances from past chimpanzee research has built the foundation of our current knowledge of HCV infection and immune response, but the need to develop alternative animal models remains urgent. In search of appropriate animal models, several non-human primates have been examined for susceptibility to HCV. For instance, treeshrews (*Tupaia belangeri*) are known to be susceptible to HCV infection [45], where it causes mild hepatitis during acute infection but does not progress to chronicity [46]. In addition, there are difficulties in the study of host responses and breeding of treeshrews, which precludes their widespread use in HCV research [26]. Neither Old World Monkeys (such as cynomolgus macaques (*Macaca fascicularis*), rhesus macaques (*Macaca mulatta*), Japanese macaques (*Macaca fuscata*), green monkeys (*Chlorocebus sabaeus*), doguera baboons (*Papio anubis*) [47] and chacma baboons (*Papio ursinus*) [48]) nor New World Monkeys (tamarin and marmoset species) have been shown to be susceptible to HCV [49,50].

Despite these limitations, these animal models have some utility, and have contributed to the comparison of HCV and monkey-tropic hepatitis viruses (see Section 3 below). Further, cell cultures of primary hepatocytes from non-human primates have been used to advance our understanding of the host barriers of HCV infection. Primary rhesus macaque hepatocytes support HCV-RNA replication and particle production, albeit at low levels [51]. Likewise, pig-tailed macaque (*Macaca nemestrina*) hepatocytes derived from induced pluripotent stem cells support the entire HCV replication cycle [52]. Although the conclusions which may be drawn from these studies have limitations due to the need to suppress innate antiviral defenses to establish infection, the results suggest that HCV infection could possibly be established in smaller non-human primates for future research.

## 3. Surrogate Models for HCV [George Baker Virus-B (GBV-B), Recent Hepaciviruses]

As it has proven very challenging to establish HCV infection in non-human species, models of HCV infection using surrogate viruses with genetic similarity to HCV are being explored: for example, George Baker virus B (GB Virus B, GBV-B) is a positive-sense single-stranded RNA virus of the family *Flaviviridae*, genus *Hepacivirus*, and is closely related to HCV. In 2013, a new hepacivirus, the guereza hepacivirus (GHV), was found in black-and-white colobus monkeys [53]. Lauck et al. identified this novel GBV-B-like virus in Old World Monkeys, with 3/9 black-and-white colobus monkeys (*Colobus guereza*) testing positive [53]. This virus, named GHV, has genetic similarity to GBV-B (48% similarity), as well as to hepaciviruses found in African bats, and in North American rodents (50% and 47% similarity, respectively).

Canine hepacivirus (CHV) was first identified in samples from dogs affected with respiratory diseases [54]. Later, horses were identified as the original host of CHV, and the virus was renamed as equine hepacivirus (EqHV). Multiple hepaciviruses have been detected in non-primate animals, and were collectively named as non-primate hepaciviruses (NPHVs) [55]. Other species such as rodents [56,57,58], bats [59], cows [60,61] and catsharks [62] were all found to be infected by NPHVs. Analyses of genetic homology and phylogenetic relatedness between GBV-B, NPHVs, and HCV are described in multiple articles and reviews [63,64,65] summarized in this section. These viruses, their hosts, and pathologies are described in Table 1.

### 3.1. Primate Viruses

In an attempt to isolate and identify the etiologic agent of non-A, non-B hepatitis, Deinhardt and colleagues inoculated various species of tamarins (*Saguinus fuscicollis*, *Saguinus nigricollis*, and *Saguinus* (*Oedipus*) *oedipomidas*) with sera from human patients with severe hepatitis [80,94]. Blood serum collected from a surgeon suffering from acute hepatitis, George Baker (GB), was found to cause hepatitis in four of the challenged tamarin species. Sera from these infected tamarins were collected and injected into naïve tamarins. This process was repeated with sera from each passage, and these serial passages of sera each also caused hepatitis. The eleventh passage of this serum was demonstrated to be infectious in most tamarins. Two viruses were identified from tamarins infected with this eleventh passage serum, and were termed George Baker viruses, GBV-A and GBV-B [81]. Subsequent studies showed that GBV-A did not cause hepatitis, but could persistently infect New World Monkeys (NWMs) without obvious disease symptoms [95]. This eleventh-passage serum from tamarins was found to be non-infectious in chimpanzees [96]. The different host tropisms and infection profiles between HCV and GBV-B may continue to prove helpful in grasping the immune evasion strategies of HCV. It has also been argued that comparing and contrasting differences in immune responses and pathogenesis between GBV-B in tamarins and HCV infection in chimps or humans may reveal correlates of immunity and factors that influence disease severity [80].

GBV-B causes hepatitis in tamarins, but the natural host has not been identified. There has not been proof as to whether this virus emerged in a modified form from the initial serial passages, from a human origin initially, or from tamarins or other primates. However, given its ability to infect NWMs, GBV-B has been used as a surrogate virus to model HCV infection. Experimental infection induces acute and chronic hepatitis in many small NWMs including red-handed tamarins (*Saguinus midas*), common marmosets (*Callithrix jacchus*) and owl monkeys (*Aotus trivirgatus*) [80,82,83,84,85,86,87]. Tamarins and marmosets, which are both small NWMs, have several advantages as an experimental animal model. The small-size of these NWMs makes them easy to handle experimentally and reduces maintenance costs when compared to larger primate models [97]. Moreover, marmosets have several key immune system similarities to humans and are suitable for experimental evaluation of innate and adaptive immune responses against viral infection [98,99,100,101]. Work with GBV-B has proven useful to understand the pathogenesis of hepatotropic viruses and to demonstrate candidate antiviral activity. Both the infection rate, and severity of acute infection following exposure to GBV-B in tamarins [88,89] and marmosets [90] is comparable to that of HCV infection in humans. Experimental GBV-B infection induces viremia and mild acute hepatitis for an average of ten weeks in tamarins and two to three months in marmosets. It is also reported that GBV-B infected-tamarins exhibit persistent viremia [82,83,91] and a recurrent increase in alanine aminotransferase (ALT) levels, with stably plateauing titers of antiviral antibodies [85]. Histopathological analyses of liver necropsy samples demonstrate diffuse and abundant fibrosis. As reported by Martin et al., tamarins inoculated with in vitro transcribed GBV-B RNA develop high-titer viremia, and one animal in this study remained viremic for over two years [83]. These features are comparable to chronic hepatitis C in humans. These results show that GBV-B can cause chronic hepatitis C-like liver disease in marmosets and tamarins.

Given that HCV and GBV are genetically similar, yet have significant differences in both host tropism and various mechanisms of infection and immune evasion, efforts have been made to create viral chimeras. A chimera of GBV-B and HCV has been utilized to overcome the limiting narrow host range of HCV. GBV-B/HCV chimeras carrying short fragments of the HCV 5′ untranslated region (UTR), hyper variable region, or p7 sequences all replicate in NWMs in vivo; however, the fitness of these viral chimeras is impaired [102,103,104,105,106]. Conversely, HCV-based chimeras harboring GBV-B E1, E2 and p6 were shown to replicate in primary marmoset hepatocytes [107]. Viral RNA was detected persistently in a tamarin after intrahepatic inoculation for more than 2 years, but this animal did not show any obvious hepatitis or increase in ALT levels. Intrahepatic injection of RNA from GBV-B/HCV chimeras harboring HCV genes E1 through p7 or HCV core through p7, however, caused chronic infection for a minimum of 40 weeks in marmosets [108]. These infected animals showed moderate elevation of aspartate aminotransferase (AST) and other clinical symptoms of viral hepatitis [108]. It was further demonstrated that serum from these viremic animals caused infection and hepatic inflammation in naïve marmosets; however, viremia was intermittent, which appeared to correlate with the induction of cellular and humoral immune responses. A GBV-B/HCV chimera carrying HCV NS2 to NS4A caused viremia in marmosets for 20 weeks, and viremia was maintained even longer under immunosuppression [109]. These results from infection studies with GBV-B/HCV chimeras remain to be validated in larger cohorts of animals, to more accurately characterize their replicative capacities, correlates of immunological protection, and virally induced pathogenesis.

### 3.2. Non-Primate Hepatic Viruses, Excluding Rodent Viruses

Over the past decade, more than 250 new viruses from non-human species have been identified in a broad range of species. This has significantly increased our knowledge of the variety, genetic diversity, and host range of hepacivirus and pegivirus species [58,92,93,110]. Overall, the genome structures of hepaciviruses and pegiviruses are conserved, with many similarities. Phylogenetic analysis confirmed that HCV is most closely related to a hepacivirus from horses, yet overall only shares approximately 50% sequence similarity [65].

Canine hepacivirus (CHV) and equine hepacivirus (EqHV), both also termed as non-primate hepaciviruses (NPHVs), have been identified as closely related new hepaciviruses found in dogs and horses in 2011 and 2012, respectively [54,55]. Kapoor and colleagues initially detected CHV RNA in 3/5 dogs, results that were confirmed in another cohort in which CHV RNA was found in livers of 5/19 animals. Burbelo and colleagues found 36/103 horse serum samples were immunoreactive to EqHV, and viral genomic RNA was found in 8/36 seropositive horse serum samples, but all dog, rabbit and deer samples were seronegative. Both groups reported that through phylogenetic analysis, CHV and EqHV were more closely related to HCV than GBV-B. In horses, EqHV causes acute mild hepatitis, but chronic infection is rare [77,78,79].

In 2013, Quan et al. determined that nearly 5% of bat samples collected worldwide were infected with hepaciviruses or pegiviruses [59]. These data suggest that bats are a considerable natural reservoir for hepaciviruses and pegiviruses, as all bats collected were healthy despite the high level of virus detected. Drexler et al. also detected seropositive bat samples as well as rodent hepacivirus (RHV) in samples collected worldwide [58]. Lauck et al. identified a novel GBV-B-like virus in Old World Monkeys, with 3/9 black-and-white colobus monkeys (*Colobus guereza*) testing positive [53]. This virus, named the guereza hepacivirus (GHV), has genetic similarity to GBV-B (48% similarity), as well as to hepaciviruses found in African bats, and in North American rodents (50% and 47% similarity, respectively).

In 2015, Corman et al. found NPHVs in sera samples from domestic Ghanaian cattle by RT-PCR [61]. The prevalence of this virus was 8.5% (9/106). Cattle hepaciviruses formed two distinct phylogenetic lineages and are not monophyletic with EqHV. Furthermore, as genetic diversity of cattle HV is low in comparison to HCV genotypes, the authors suggest that human modified-ecology has interfered with hepaciviral evolution in domestic cattle. In Germany, Baechlein et al. detected cattle HV in 1.6% (5/320) serum samples of cattle [60]. This virus was detected in liver, lung, lymph node, and serum samples. Liver samples showed the highest replication rates, indicating a pronounced hepatic tropism. However, there were no clinical signs nor signs of liver damage, such as increased liver enzyme levels, detected.

Most recently, Shi et al. discovered a novel hepacivirus in 2/56 liver tissue samples from catsharks (*Proscyllium habereri*), a well as five segmented jingmenviruses and twelve kinds of Flavi-like viruses from arthropod and vertebrate host species [62]. This study extends the known host ranges of hepacivirus and pegiviruses from mammals to other vertebrates. At present, it is unclear whether any of these viruses in their respective natural host species would serve as potentially suitable surrogate models for HCV infection.

### 3.3. Rodent Viruses

Kapoor and colleagues described the naturally occurring rodent hepacivirus RHV for the first time in 2013 [56]; various other RHVs were subsequently isolated around the world. In 2014, an RHV isolated from urban rats (Norway rats, *Rattus norvegicus*) in New York city was identified and named NrHV [57]. This virus was found to be capable of productive infection in laboratory mice and has been used as a surrogate model of HCV for use in infection of mice and rats. This virus shows amino acid divergence of approximately 70% from HCV, which is similar to the divergences between HCV and GBV-B, and substantially greater than that of HCV and EqHV [56]. Trivedi et al. demonstrated that NrHV (also named as RHV-nr-1) can cause persistent infection in immunocompetent rats [92]. They found that the 5′UTR of RHV-nr-1 had two conserved microRNA-122 (miR-122) seed sites, which are important for HCV replication and hepatotropism in humans. These sites are required for RHV-nr-1 replication and persistence in rats. In parallel, it was shown that laboratory mice infected with RHV-nr-1 exhibit viremia and HCV-like hepatitis [93]. Whereas immunocompetent mice cleared the virus within 3–5 weeks, immunocompromised mice develop persistent infection lasting for longer than 40 weeks. These studies also defined critical roles of CD4^+^ T cells in clearance of this virus.

In efforts to make RHV-nr-1 more genetically tractable, Wolfisberg et al. established an in vitro culture system that is highly useful for reverse genetic analysis of this virus [110]. An RHV-nr-1 replicon was established and shown to replicate in rat hepatoma cell lines. Analogously to HCV, adaptive mutations—here in NS4B and NS5A—enhanced viral replication in cell culture. Using this replicon, it was shown that the RHV-nr-1 NS3-4A protease cleaves human mitochondrial antiviral-signaling protein (MAVS), again showing similarity to HCV and indicating compatibility and potential for use as an HCV surrogate for study. In addition, the RHV-nr-1 replicon can be inhibited by the HCV polymerase inhibitor sofosbuvir, HCV NS5A-targeting antivirals, and a miR-122 antagonist, highlighting some critical similarities between HCV and RHV-nr-1.

Despite its considerable genetic and antigenic divergence from HCV, the RHV surrogate model holds clear potential utility in vaccine development. Hartlage and colleagues examined immunological responses to vaccination with a recombinant adenovirus vector (AdV) expressing hepacivirus non-structural proteins (NS3-5B) in rats [66]. Non-immunized rats infected with RHV-nr-1 develop chronic viremia associated with limited virus-specific T-cell responses. In particular, non-functional CD8^+^ T cells expand and interfere with viral clearance [66]. In comparison, immunization with the AdV-NS3-5B experimental vaccine primed antigen-specific CD8^+^ and CD4^+^ T cells to produce IFNγ and TNFα in liver, resulting in elimination of virus after challenge in 6/9 mice [66]. In addition, transient CD8^+^ T-cell depletion in vaccinated rats resulted in prolonged infection, followed by clearance within 84 weeks, while CD4^+^ T cell depletion resulted in development of chronic infection, with viremia lasting at least 126 weeks. These studies indicate that both CD8^+^ and CD4^+^ T cells play pivotal roles in the clearance of this hepacivirus [66]. Subsequent immunophenotyping by Hartlage and colleagues showed that the virus-specific and non-functional CD8^+^ T cells, which proliferated considerably in the liver, acquired an exhausted phenotype as evidenced by the upregulation and sustained expression of programmed cell death-1 (PD-1) [111]. Immune dysregulation during persistent RHV-nr-1 expression was further substantiated by the fact that virus-specific CD8^+^ T cells failed to form any immunological memory phenotype including the expression of CD127 [111]. These features are commonly observed in persistent HCV carriers.

Another vaccine candidate, simian adenovirus encoding a genetic immune enhancer (truncated shark class II invariant chain) fused to RHV NS3-5B, was evaluated by Atcheson et al. using Sprague-Dawley rats infected with RHV-nr-1 [112]. Vaccination induced CD8^+^IFNγ^+^ T cells; of particular interest, this population reacted with NS4 in the blood, spleen and liver. Viremia after challenge in immunized rats was inhibited, with 42% of rats clearing infection within 6–8 weeks. Immunization efficacy was enhanced both by a high single dose, as well as following with a boost immunization. 67% of rats eliminated the virus when challenged after a high single dose, and 100% of rats cleared the virus when challenged following boost immunization. These immunization groups showed high recall of CD4^+^IFNγ^+^ T cells and CD8^+^IFNγ^+^ T cell responses, particularly against NS4. This population of liver-infiltrating CD8^+^IFNγ^+^ T cells remained at a high level until approximately 150 days after infection [112]. Rats with undetectable viremia exhibited a significantly higher CD8^+^IFNγ^+^ T cell response than rats with high viremia, but response changes in CD4^+^IFNγ^+^ T cell levels were not statistically significant between the groups. In addition, a vector encoding structural proteins alone showed no efficacy; however, combined vaccination with a vector encoding NS3-5B showed additive effects. This indicates that protection is associated with CD8^+^IFNγ^+^ T cell responses against NS4. Cellular immunity is more critical than humoral immunity for control of infection, although humoral immunity and cellular immunity may work in conjunction to control infection, as suggested by the results that mixed immunization against both structural and non-structural antigens results in enhanced protection [112].

Overall, these studies show that RHV-nr-1 in rats may have some utility for assessing general principles of anti-hepacivirus immunity. Given the considerable genetic differences between HCV and RHV-nr-1, however, it remains unclear how readily data obtained with RHV-specific reagents or vaccine candidates can be extrapolated to HCV infection in humans.

## 4. Murine Models of HCV Entry and Infection

Mouse models have proven ideal for research of human disease in many fields. Genetic accessibility, quick generation time, low maintenance costs, and well-established sources and protocols relative to other potential vertebrate animal models make them a preferred workhorse in many areas of research. As discussed previously, because of the inability of HCV to infect mice [113,114,115], many alternative approaches have been taken to use mice productively in HCV research. In addition to the study of rodent hepatotropic viruses, which serve as surrogates for HCV infection (discussed in Section 3.3), mice have been used to study HCV infection by transgenic expression of the partial or entire HCV genome along with specific human factors known to interact with the virus and/or xenotransplantation of human hepatocytes and immune cells. Each of these approaches has contributed to our current knowledge of HCV infection in different ways, and many approaches continue to hold promise to advance the field and our ability to develop treatments and prophylactics for HCV. A brief outline of the models still in use is highlighted in Table 1.

### 4.1. HCV Transgenic Mice

Due to early difficulty in establishing surrogate models of HCV infection in mice, transgenic technologies were an important advancement for HCV research. When transgenic experiments became feasible in mice, full length HCV, as well as specific HCV proteins, were expressed in mice to investigate the specific effects of HCV proteins in vivo [116,117]. Use of HCV-expressing mice, with various additional transgenic manipulations of host and viral factors, allowed for determination of specific localization changes of HCV proteins, such as the core protein, which can increase the rates of steatosis and HCC in mice up to those seen in patients with chronic HCV infection [116,117,118]. Congruent with epidemiological data, rates were higher in older male mice with long-term exposure when compared to other age and sex groups. One key discovery from subsequent work was that HCV-expressing mice exhibited oxidative stress that lead to liver disease and HCC in the absence of canonical immune responses, ultimately leading to chronic inflammation [119]. Additionally, use of these HCV-expressing mice helped establish that HCC develops at higher rates in HCV patients, and along a faster timeline, due to HCV proteins that cause stress and promote liver disease even without all of the genetic changes that are normally necessary for development of HCC [120]. As such, these HCV-expressing models proved critical for our understanding of how long-term exposure to HCV proteins can induce immune responses, inflammation, and HCC development.

Most recent studies and ongoing research have focused on other models that more accurately recapitulate the known mechanisms of HCV infection and development of chronicity, so as to probe the interactions of HCV with the immune system during early stages of infection and onwards. Despite this large shift in preferred model systems, the importance of the HCV-expressing mouse models for our understanding of chronic HCV infection remains significant. It should be noted, however, that the mouse immune system is tolerized to the HCV protein products in such models and thus the inflammatory environment in which pathogenic phenotypes emerge is arguably very different from the situation observed in humans.

### 4.2. Genetically Humanized Mouse Models

As stated before, mice are not naturally susceptible to HCV infection. In 2009, it was shown that out of the four canonical HCV entry factors—CD81, scavenger receptor type B class I (SCARB1), and the tight junction proteins claudin 1 (CLDN1) and occludin (OCLN)—only human CD81 and OCLN need to be of human origin in order to facilitate HCV glycoprotein-mediated entry into mouse, hamster or primate cells [67]. Subsequent work expressing human CD81 and OCLN in mice allowed HCV entry into murine hepatocytes [67]. Later, knock-in mice carrying humanized extracellular loop domains of CD81 and OCLN were shown to also support HCV entry into mouse liver cells [121]. This also validated prior data demonstrating that the species tropism defining residues of these proteins all lay within their second extracellular loops [68,122]. Identification of CD81 and OCLN as the minimal set of human-specific factors spurred efforts to genetically adapt HCV to enter using the murine orthologs of these factors. This directed adaptation culminated in the identification of three mutations in the HCV envelope proteins [69], which together result in facilitated uptake of designed murine-tropic HCV viruses, termed mtHCV or Jc1/mCD81 into mouse hepatocytes both in vivo and in vitro without any transgenic expression of human factors in these mice [123]. These immunocompetent HCV entry models have found some utility in preclinical testing of HCV entry inhibitors [71], treatment with passively infused neutralizing antibodies [124], and vaccine candidates based on inducing humoral immunity [113,125].

Even after crossing the entry barrier, however, replication and infectious particle production are still blocked in mouse hepatocytes, highlighting the difficulty of producing effective virus infection in cell types that do not natively sustain this infection. This work followed the discovery of human factors that allowed entry and replication of HCV in murine cell culture [70,126], stressing the importance of cell-culture and animal model experiments working in tandem to explore newly discovered host–virus interactions. Later it was established that the entire HCV replication cycle could be recapitulated in mice by crossing humanized CD81- and OCLN- expressing mice with mice deficient in STAT1 or IFNαβ receptor [127]. However, even when blunting innate immunity in mice, HCV RNA replication is still very low, suggesting that other genetic incompatibilities exist that hinder HCV replication in mouse cells. One such factor may be cyclophilin A (CypA), an essential cofactor in HCV replication. The inability of mouse CypA to facilitate HCV replication lead to studies comparing cyclophilin A orthologs between species as a contributor to host range determination of HCV [128]. Notably, overexpression of human CypA in mouse hepatoma cells engineered to express OCLN, CLDN1, SCARB1, and CD81, with or without miR-122 [129] and SEC14L2 [130], only marginally increased HCV RNA replication [128]. This suggests that other unidentified positive and negative regulators exist that modulate efficient HCV replication in mouse cells

### 4.3. Humanized Xenotranslation Models: Human Liver and Immune Cell Transplantation

Given the robust human hepatotropism of HCV, efforts have been made to engraft human hepatocytes into suitable xenorecipients. Specific lines of immunodeficient mice have been developed to allow xenotransplantation of tissues or cell types of interest [131]. These have been used to great advantage in HCV research. Mice which can be successfully engrafted with both human liver tissue and immune system cells have been the subject of numerous reviews [26,115,132,133,134]. The earliest use of chimeric engrafted mice in HCV research used severe combined immunodeficient (SCID) mouse bone marrow cells engrafted into irradiated beige/nude/x-linked immunodeficient mice (BNX mice) [72]. These mice then had liver segments from chronically infected HCV patients implanted into their abdominal cavities to study HCV viremia and infection maintenance. HCV RNA was detected in serum from these mice, as well as in that of mice implanted with normal human liver samples, which were incubated with HCV prior to implantation. This proved an interesting and useful early model of xenotransplantation in the study of HCV infection in mice, but the difficulty of obtaining sufficient supply of HCV-infected or normal adult human liver samples prevents this from being a widely used major model system. Furthermore, the ongoing utility of this model is limited by its inability to probe early infection stages and immune responses.

Pioneering work by Mercer, Tyrell and Kneteman showed that immunodeficient mice transgenically expressing the hepatotoxic urokinase type plasminogen activator (uPA) under the control of an albumin (Alb) promoter can be robustly engrafted with human hepatocytes [73]. The resulting human-liver chimeric mice sustain high level HCV infection and have since been used for testing a plethora of antiviral therapies. Subsequently, similar results have been obtained in a variety of liver injury models [74,132,133], including the above mentioned Alb-uPA [73,75] mice as well as fumarylacetoacetate hydrolase knockouts (FAH^−/−^ mice) [76,124], mice expressing a fusion protein of the FK506 binding protein and caspase 8 under control of the albumin promoter (AFC8 mice) [74], mice expressing uPA under the promoter for major urinary protein (MUP-uPA mice) [135], and mice expressing herpes simplex virus thymidine kinase (HSV-TK) [136]. These human-liver chimeric mice support infection with a variety of hepatotropic pathogens, including HBV, HCV, HDV, and most recently HEV [73,76,124,137,138,139,140,141,142,143]. These mice have been used to study innate host responses to many hepatitis viruses, including HCV, and for testing the efficacy of novel therapeutic regimens; however, the highly immunocompromised status of these human-liver chimeric mice precludes the study of immune-mediated pathogenesis by HCV.

To enable analysis of human immune responses to HCV, protocols are being designed to co-engraft mice with human hepatocytes and components of a human immune system (HIS). Double humanization of both the liver and immune system has been achieved with human hematopoietic stem cells (HSCs) and either adult [144,145,146,147] or fetal hepatocytes [74,148,149]. Maturation of fetal hepatoblasts by exogenous administration of human oncostatin M (OSM) considerably boosted human hepatic chimerism, affirming that less mature hepatic cells do not proliferate in response to liver injury in these xenorecipients. Dually engrafted mice can support HCV and HBV infection [74], and studies have shown that viral infection triggers activation of the engrafted HIS [147], in particular natural killer (NK) cells [148] and M2 macrophages [150], and leads to some virally induced histopathology [150].

Undoubtedly, humanized mice cannot yet perfectly mimic the complex situation of chronic HCV in patients, and only some very important aspects of it have been recapitulated so far. Future refinements will have to focus on improving the limited functionality of the engrafted HIS. Numerous strategies to do this have been proposed (reviewed in [151,152]), as several human cell lineages remain underrepresented in part due to the orthologs of non-redundant cytokines, which exhibit limited biological cross-reactivity. It was previously shown that selective expansion of under-represented cell types, such as dendritic cells, NK cells and granulocytes, leads to markedly improved immune responses to the yellow fever virus (YFV) vaccine, akin to those observed after administration of YFV vaccines to patients [153]. Additionally, the development of functional adaptive immune responses is limited by the lack of human leukocyte antigen (HLA) gene expression. Expressing a human MHC class I allele has multiple benefits, as it allows for more faithful development of CD8^+^ T cells in the thymus, enables recognition of (viral) antigens in peripheral tissues by human CD8^+^ T cells and facilitates tracking of antigen-specific CD8^+^ T cells with MHC multimers, as previously shown for Epstein-Barr virus and dengue virus [154,155,156].

A shortcoming of conventional humanized mouse models is the lack of human MHC class II expression, which may result in CD4^+^ T cell lineage disfunctions. It has previously been suggested that expression of a human MHC class II molecule, HLA-DR4, partially improves the development of functional human T and B cells [157,158]. Previous work characterized adaptive immune responses to adenovirus infections in humanized HLA-A*0201 and HLA-DRB*01 doubly transgenic mice and found statistically significant clearance of viral antigens from the liver [159].

Currently, most studies employ antigen-inexperienced or “naïve” mice, which do not take into account how immunological history shapes immunity. Exposing mice to multiple vaccines or infections that are acutely resolved may be a valuable approach to more accurately mimic human immune responses. Co-engraftment of improved xenorecipient strains with additional HSC donor-matched human tissues, such as liver, thymus and/or lymph nodes, could also significantly augment the immune response. Such co-engraftments could enhance T and B cell selection, intra-hepatic T cell priming [160] and liver-mediated secretion of key human-immune components [161]. Finally, engraftment of second-generation humanized mice with a human-like microbiome represents another valuable approach to enhance immunity, as recently suggested [162].

## 5. Future Directions in HCV Animal Model Development

As HCV research progresses, there will be continued interest in understanding protective immunity and developing potential vaccines. These efforts will likely rely on both genetically humanized and dually engrafted xentotransplanted human-chimeric mouse models for initial discovery and screening approaches. Advances in understanding host immune responses may continue to come from surrogate models of HCV infection in rodent hepaciviruses and non-human primate models with GBV-B, but humanized rodent models are likely to receive continued interest for discovery efforts due to their more direct application to human infection. As we advance our understanding of specific factors necessary for HCV to complete its replication cycle and produce infectious particles, genetically humanized models will clearly progress. This will prove useful in deciphering the roles of each factor alone and in combination with others. Regardless of the model system used, animal models will doubtless prove to be incredibly important in continuing efforts to develop an effective prophylactic HCV vaccine, as well as alternative treatment regimens.

## Figures and Tables

**Table 1:** Hepatitis C virus (HCV) and related hepacivirus infections, organized by species where infection is detected. Shaded rows are those being currently used and developed for animal model studies.

Summary of HCV and Related Hepaciviruses: Permissive Species, Pathology, and Use as Models of Infection				
Virus	Host	Pathology	Notes	Selected References
HCV	Human	Acute infection/hepatitis Chronic infection/hepatitis, HCC	Little acute phase data	[1,2,22,23]
HCV	Chimpanzee	Acute infection/hepatitis Chronic infection/hepatitis, rarely HCC	Lower chronic rate than human cases, use of chimpanzees is banned in most countries; no more federal funding for chimp research in the US	[27,28,29,30,31]
HCV	Treeshrew	Acute infection/mild hepatitis, Chronic infection?	Difficult breeding	[45,46]
HCV	Mouse: Genetically humanized	Entry Permissive. Only low level replication in immunocompromized mice; limited pathology	Some infection experiments also involve viral adaptation to murine orthologs of identified factors	[66,67,68,69,70]
HCV	Mouse: Humanized via xenotransplantation	High level of sustained infection and viremia; no overt disease in singly engrafted human liver chimeric mice	Immune activation and evidence for virally induced liver pathology in dually (liver / immune system) engrafted mice	[71,72,73,74,75,76]
EqHV/CHV	Horse, Dog	Horse: Acute infection/mild hepatitis, rare chronic infection, no chronic hepatitis		[54,55,77,78,79]
GBV-B	New World Monkeys	Acute infection/mild hepatitis rare chronic infection/hepatitis	Unstable chronic infection rate, applicable chimeric virus	[80,81,82,83,84,85,86,87,88,89,90,91]
RHV	Mouse, Rat	Acute infection/mild hepatitis, chronic infection/hepatitis	High accessibility, applicable gene-edited mice/rats	[56,57,92,93]
BHV	Bat	No overt clinical signs of disease	Natural reservoir?	[59]
GHV	Colobus Monkey	No overt clinical signs of disease		[53]
Cattle HV	Cow	No overt clinical signs of disease		[60]
Wenling Shark Virus (WLSV)	Graceful catshark	No overt clinical signs of disease		[62]

## Abbreviations

MDPI	Multidisciplinary Digital Publishing Institute
DOAJ NJ	Directory of Open Access Journals New Jersey
HCV	Hepatitis C Virus
HCC NHP GBV NPHV CHV EqHV NWM ALT AST UTR RHV RHV-nr-1 NrHV MAVS AdV IFNγ IFNab TNFβ HLA MHC CLDN1 OCLN STAT1 CypA SCARB1 miR-122 SEC14L2 mtHCV SCID BNX uPA PD-1 HBV HDV HEV YFV HIS OSM	Hepatocellular Carcinoma Non-Human Primates George Baker Virus Non-Primate Hepacivirus Canine Hepacivirus Equine Hepacivirus New World Monkeys Alanine Aminotransferase Aspartate Aminotransferase Untranslated Region Rodent Hepacivirus Rodent Hepacivirus isolated from *Rattus norvegicus*-1 Norway Rat Hepacivirus Mitochondrial Antiviral Signaling Protein Adenovirus Vector Interferon Gamma Interferon Alpha-B Tumor Necrosis Factor-Beta Human Leukocyte Antigen Protein Major Histocompatibility Complex Claudin-1 Occludin Signal Transducer and Activator Of Transcription 1 Cyclophillin A Scavenger Receptor Class B Member 1 MicroRNA 122 SEC14 Like-2 Murine-Tropic Hepatitis C Virus Severe Combined Immunodeficient Mice Beige/Nude/X-Chromosome-Linked Immunodeficient Mice Urokinase Type Plasminogen Activator Programmed Cell Death Protein 1 Hepatitis B Virus Hepatitis Delta Virus Hepatitis E Virus Yellow Fever Virus Humanized Immune System Oncostatin M
